# Endoscopic ultrasound staging in patients with gastro-oesophageal cancers: a systematic review of economic evidence

**DOI:** 10.1186/s12885-019-6116-0

**Published:** 2019-09-09

**Authors:** Seow Tien Yeo, Nathan Bray, Hasan Haboubi, Zoe Hoare, Rhiannon Tudor Edwards

**Affiliations:** 1 Centre for Health Economics and Medicines Evaluation (CHEME), Bangor University, Ardudwy, Normal Site, Holyhead Road, Bangor, Gwynedd LL57 2PZ UK; 2 Cancer Biomarkers Group, Swansea University, Singleton Park, Swansea, SA2 8PP UK; 3 North Wales Organisation for Randomised Trials in Health and Social Care (NWORTH), Bangor University, Y Wern, Normal Site, Holyhead Road, Bangor, Gwynedd LL57 2PZ UK

**Keywords:** Costs, Effects, QALYs, Economic review, Endoscopic ultrasound, EUS staging, Staging techniques, Gastro-oesophageal cancers

## Abstract

**Background:**

The sensitivity of endoscopic ultrasound (EUS) in staging gastro-oesophageal cancers (GOCs) has been widely studied. However, the economic evidence of EUS staging in the management of patients with GOCs is scarce. This review aimed to examine all economic evidence (not limited to randomised controlled trials) of the use of EUS staging in the management of GOCs patients, and to offer a review of economic evidence on the costs, benefits (in terms of GOCs patients’ health-related quality of life), and economic implications of the use of EUS in staging GOCs patients.

**Methods:**

The protocol was registered prospectively with PROSPERO (CRD42016043700; http://www.crd.york.ac.uk/PROSPERO/display_record.php?ID=CRD42016043700). MEDLINE (ovid), EMBASE (ovid), The Cochrane Collaboration Register and Library (including the British National Health Service Economic Evaluation Database), CINAHL (EBSCOhost) and Web of Science (Core Collection) as well as reference lists were systematically searched for studies conducted between 1996 and 2018 (search update 28/04/2018). Two authors independently screened the identified articles, assessed study quality, and extracted data. Study characteristics of the included articles, including incremental cost-effectiveness ratios, when available, were summarised narratively.

**Results:**

Of the 197 articles retrieved, six studies met the inclusion criteria: three economic studies and three economic modelling studies. Of the three economic studies, one was a cost-effectiveness analysis and two were cost analyses. Of the three economic modelling studies, one was a cost-effectiveness analysis and two were cost-minimisation analyses. Both of the cost-effectiveness analyses reported that use of EUS as an additional staging technique provided, on average, more QALYs (0.0019–0.1969 more QALYs) and saved costs (by £1969–£3364 per patient, 2017 price year) compared to staging strategy without EUS. Of the six studies, only one included GOCs participants and the other five included oesophageal cancer participants.

**Conclusions:**

The data available suggest use of EUS as a complementary staging technique to other staging techniques for GOCs appears to be cost saving and offers greater QALYs. Nevertheless, future studies are necessary because the economic evidence around this EUS staging intervention for GOCs is far from robust. More health economic research and good quality data are needed to judge the economic benefits of EUS staging for GOCs.

**PROSPERO Registration Number:**

CRD42016043700.

**Electronic supplementary material:**

The online version of this article (10.1186/s12885-019-6116-0) contains supplementary material, which is available to authorized users.

## Background

Gastro-oesophageal (oesophageal or gastric, or both) cancers (GOCs) are one of the most common cancers in the UK with approximately 16,000 people diagnosed in 2015 [1, 2]. Oesophageal and gastric cancers were the seventh and fourteenth most common cause of cancer death respectively in the UK in 2016, as shown from the latest available statistics reported by the Cancer Research United Kingdom (CRUK) [1, 2]. It is estimated that a total of around 12,500 people died from these cancers in 2016 – that is 34 deaths per day [1, 2]. Accurate staging of GOCs is vital for determining prognosis and planning appropriate treatment. Accurate staging in the management of GOCs will not only help avoid unnecessary surgical interventions but also will ultimately help reduce the financial pressure on the NHS, which is particularly important given the limited resources available to cancer services and the growing incidence of GOCs [3].

Accurate staging of GOCs can be achieved by a combination of investigative techniques. The techniques used for staging GOC include computer tomography (CT), endoscopic ultrasound (EUS), positron emission tomography (PET) and adjuncts to staging include magnetic resonance imaging (MRI), bronchoscopy, laparoscopy and trans-abdominal ultrasound [4]. CT has been recommended for use at initial staging assessment to determine whether the cancer cells have spread from the primary site of its origin into new areas of the body (i.e. metastasis); but in the absence of metastatic disease, EUS has been advocated as the preferred technique for the assessment and prediction of operability [4]. This is due to the fact that EUS is superior to CT for local regional staging of oesophageal and gastric tumours [4].

Studies and guidelines for the management of oesophageal and gastric cancer have reported that EUS has superior tumour invasion (T) and loco-regional nodal (N) staging ability over CT and PET given its sensitivity, particularly for detection of regional lymph node metastases, although the complementary nature of these investigative techniques must be recognised [5–10]. The sensitivity of EUS for staging of GOC has been widely evaluated; however, the economic evidence of EUS staging in the management of GOC patients is scarce. Furthermore, the effectiveness and cost-effectiveness of EUS staging of GOC had not been assessed, particularly in the form of randomised controlled trials (RCT), until the establishment of “COGNATE” trial - a HTA-funded RCT UK study [11].

Given that the economic evidence of EUS for staging of GOC is scant, conducting a systematic review of the economic evidence on EUS staging in patients with GOC is therefore important. It not only gives a meaningful evidence-based insight, from an economic perspective, for researchers and clinical experts in this field but also healthcare commissioners. In view of that, this systematic review aimed to examine all economic evidence (not just from RCTs) of the use of EUS staging in the management of patients with GOC. Systematic reviews of economic evaluations review studies that evaluated both the effectiveness in terms of health effects (usually measured as life-years gained (LYGs) or quality-adjusted-life-years (QALYs), accounting for the quality-of-life outcomes) and cost of the alternative interventions assessed. Economic evaluation is performed by undertaking either a cost-effectiveness analysis (CEA), cost-utility analysis (CUA), cost-consequences analysis (CCA), cost-benefit analysis (CBA) or cost-minimisation analysis (CMA). When clinical outcome expressed in natural units (e.g. LYGs, lives saved, improvement in pain score etc) are used as health effects in an economic analysis, this is often referred to as CEA with its parameter of interest being called incremental cost-effectiveness ratio (ICER). Whereas, when QALY, a common unit, is used as health effect in an economic analysis, then this is often referred to as CUA though CEA is preferred by some authors and the resulting parameter of interest is called incremental cost-utility ratio (ICUR). The ICER/ICUR is then compared with the official or approximate willingness to pay (WTP) ceiling ratio for a unit of effect, that is, threshold used for decision making. CCA reports costs and outcomes in disaggregated form for each alternative [12]. CBA converts clinical outcomes into monetary units so that a net benefit (or cost) can be estimated [12]. CMA measures which alternative has the least cost, this method is only applied when the outcomes of alternative interventions have been proven to be equivalent. The protocol of this systematic review was registered prospectively with PROSPERO, an international prospective register of systematic reviews (Registration number 2016:CRD42016043700; http://www.crd.york.ac.uk/PROSPERO/display_record.php?ID=CRD42016043700) [13]. This paper offers a review of economic evidence on the costs, benefits (in terms of GOC patients’ health-related quality of life), and economic implications of the use of EUS for staging GOC patients.

## Methods

This review was carried out and reported in accordance with the published updated Preferred Reporting Items for Systematic Reviews and Meta-Analyses (PRISMA) guidelines [14, 15].

### Searches and study selection

Searches for this systematic review were conducted using a range of electronic databases: MEDLINE (ovid), EMBASE (ovid), The Cochrane Collaboration Register and Library (including Cochrane Central Register of Controlled Trials (CCRCT), Cochrane Reviews, Database of Abstracts of Reviews of Effects (DARE), Health Technology Assessment (HTA), British National Health Service Economic Evaluation Database (NHS EED), Cochrane Methodology Register (CMR)), CINAHL (EBSCOhost), Web of Science (Core Collection). Searches were restricted to publications from the last 20 years (1996–2016) as per the registered protocol on PROSPERO (Registration number 2016:CRD42016043700) [13]. To ensure that the review was as up-to-date as possible, the searches were re-run on all databases to cover 2016–2018 (search update on 28/04/2018).

In order to ensure a comprehensive search was achieved and any relevant research had not been missed, online searches were also conducted through the following internet search engines and appropriate websites to identify grey literature, reports, ongoing and unpublished studies from conference papers and abstracts: Google, Google Scholar, Department of Health (DoH), National Institute for Health and Clinical Excellence (NICE), National Institute for Health Research (NIHR) Journals Library, NIHR UK Clinical Trials Gateway, The National Cancer Research Institute (NCRI), Cancer Research Wales (CRW), Wales Cancer Research Centre (WCRC), Welsh Government (WG), Health and Care Research Wales (HCRW), CRUK and other relevant charitable organisation websites.

The reference lists of papers that were included in the review were searched for further publications that had not been identified in the electronic searches. Contacts with study authors were made to locate further relevant literature and publications.

Guided by the review objectives, the search terms as shown in Table 1 were developed using the PICO framework [16, 17]. The PICO framework was utilised to help shape, design and construct the search process to identify all relevant published and unpublished materials from various sources. Titles, abstracts and full-text papers were searched for using these search terms.

**Table 1:** Search terms by category, guided by PICO framework, for the systematic review

No.	Search Term Category	Search Terms
1.	Disease	neoplas* OR
		cancer*OR
		carcin* OR
		tumo* OR
		adenocarcinoma* OR
		squamous cell carcinoma* OR
		malig* OR
		metasta*
		AND
2.	Type of disease	gastro* OR
		oesophag* OR
		esophag* OR
		gastro-oesophag* OR
		gastro-esophag* OR
		gastroesophag* junction* OR
		gastro-esophag* junction* OR
		gastrooesophag* junction* OR
		gastro-oesophag* junction* OR
		esophagogastric junction* OR
		esophago-gastric junction* OR
		oesophagogastric junction* OR
		oesophago-gastric junction* OR
		oesophageal squamous cell carcinoma* OR
		esophageal squamous cell carcinoma* OR
		gut* OR
		gullet* OR
		food pipe OR
		stomach OR
		upper GI OR
		upper-GI OR
		upper gastrointestin* OR
		upper-gastrointestin* OR
		upper digestive tract* OR
		upper-digestive tract* OR
		intraepithelial OR
		intramucosal OR
		node* OR
		nodal
		AND
3.	Intervention	endosono* OR
		EUS OR
		endoscopic ultraso* OR
		endoscopic-ultraso* OR
		EUS-FNA OR
		EUS-fine needle aspiration OR
		EUS fine-needle aspiration OR
		Endosonography-guided FNA OR
		Endoscopic ultrasound-fine needle aspiration OR
		Endoscopic ultrasound-guided fine needle aspiration OR
		Endoscopic ultrasound-guided fine-needle aspiration OR
		Endoscopic-ultrasound-guided fine-needle aspiration OR
		Endoscopic ultrasound guided fine needle aspiration OR
		Echoendoscop* OR
		Echo-endoscop*
		AND
		Staging OR
		Preoperative staging OR
		Pre-operative staging
		AND
4.	Outcome	econom* OR
		health economics OR
		economic evaluation OR
		cost-effective* OR
		cost effect* OR
		cost utility OR
		cost-utility OR
		cost-conseq* OR
		cost conseq* OR
		cost-benefit OR
		cost benefit OR
		cost-minimisation OR
		cost minimisation OR
		cost-minimization OR
		cost minimization OR
		cost* OR
		cost* analys* OR
		unit cost OR
		unit-cost OR
		unit-costs OR
		unit costs OR
		drug cost OR
		drug costs OR
		hospital costs OR
		health-care costs OR
		health care cost OR
		medical cost OR
		medical costs OR
		cost* efficacy* OR
		cost* analys* OR
		cost* allocation* OR
		cost* control* OR
		cost* illness* OR
		cost* affordable* OR
		cost* fee* OR
		cost* charge*
		economic model* OR
		markov* OR
		budget* OR
		healthcare economics OR
		health care economics OR
		cost analys* OR
		health-care cost* OR
		health care cost* OR
		hrqol OR
		Health related quality of life OR
		health-related quality of life OR
		quality-adjusted life year* OR
		quality adjusted life year* OR
		qaly OR
		Quality of life OR
		quality-of-life OR
		QoL

The search strategy for each of the five electronic databases was developed, checked and tested by an information specialist before finalising the search terms; this process was informed by the search strategy of a wider evidence synthesis that includes a systematic review of non-economic studies of treatments for resectable adenocarcinoma of the stomach, gastro-oesophageal junction and lower oesophagus [18]. An example of search strategy used in the Medline Ovid database is as shown in the additional file (see Additional file 1).

### Inclusion and exclusion criteria

Table 2 presents the inclusion and exclusion criteria, using the economic evidence review design framework outlined in the University of York Centre for Reviews and Dissemination (2009) [12]: Population, Interventions, Comparators, Outcomes, and Type of Evidence. Due to resources constraints, only studies written in English were included. This includes international studies that have been translated or written in English.

**Table 2:** Inclusion and exclusion criteria for the systematic review

	Inclusion Criteria	Exclusion Criteria
Population	All adults (aged 19 and above) who had cancer (i.e. localised tumour) of the oesophagus, stomach or gastro-oesophageal junction; free of metastatic disease.	Population aged below 19 years and had metastatic oesophageal, gastro-oesophageal or gastric cancer.
Interventions	Use of endoscopic ultrasound (EUS) (also known as endosonography, echoendoscopy) staging in patient with oesophagus, gastro-oesophageal and gastric cancer.	Use of endoscopy only or ultrasound only, and use of EUS for non-cancer staging purposes e.g. treatment of cancer
Comparators	Standard staging algorithm e.g. trans-abdominal ultrasound scan, computed tomography (CT) scan. Partial economic evaluations, when no formal comparator was used, were included.	
Outcomes	All relevant full economic evaluation studies outcomes including (but not be restricted to) cost per QALY and cost per life-year gained; All other relevant economic outcomes including (but not be restricted to) resource use, direct and indirect costs, incremental benefits e.g. quality-adjusted survival or quality-adjusted life years (QALYs), health-related quality of life, cancer-specific quality of life and utility gained – this includes partial economic evaluation studies outcomes, which costs or consequences alone of a single intervention (e.g. EUS staging of GOC) were described, were included.	All outcomes unrelated to economic evidence of EUS staging of the oesophagus, gastro-oesophageal junction or gastric cancer.
Type of Evidence	Full economic evaluation evidence (i.e. cost-effectiveness, cost-utility and cost-benefit analyses) related to EUS staging of oesophageal, gastro-oesophageal junction and gastric cancer were considered. Other economic studies that contain partial economic evaluation or no evaluation context (e.g. cost analyses, cost-description studies, cost-outcome descriptions, budgetary studies, outcome-description studies in terms of utility gained, health-related quality of life and cancer-specific quality of life measures such as QALYs and FACT-G score) were also considered. Economic evaluation studies conducted alongside RCTs, non-RCTs, quasi-experimental trials, epidemiological research, cohort studies, and modelling studies were considered.	Non-research studies such as editorials, case reports or other descriptive studies.
General	Language – English. Years – 1996-2016 and 2016–2018	Language – Not written or translated into English. Years – Before 1996.

### Data extraction

Titles and abstracts of all studies identified were screened and assessed for relevance against the inclusion criteria by two independent reviewers (STY and NB). The inclusion or exclusion of each study was checked and confirmed. All potentially relevant full-text papers were then obtained and screened against the inclusion criteria, with disagreements resolved through discussion until agreements were achieved collectively. Disagreements occurred when for example the reviewers had different views on whether a retrieved paper should be included in the review.

Following screening, relevant information from all full-text papers included in the review were extracted by the primary reviewer (STY) using an adapted standardised form [12], and checked by the second reviewer (NB). Two adapted standardised forms were developed and used for data extraction – one for economic studies and another for economic modelling studies.

### Quality assessment

The quality of all full-text papers included in the review were assessed and rated independently by the two reviewers using the Critical Appraisal Skills Programme (CASP) economic evaluation checklist [19] tool for economic studies and the Philips et al’s economic modelling checklist [20] tool for economic modelling studies. The papers were critically appraised to assess to what extent the content of these papers complied with the criteria of good practice in economic evaluation and if there was any obvious bias. Disagreements between the reviewers were resolved through discussion until agreements were achieved collectively. Disagreements occurred when for example the reviewers had different score on an included paper.

### Data synthesis

All studies included in the review were summarised and compared across studies in a narrative form to answer the review objectives. The aims, methods, and results of the studies reviewed were synthesised narratively. This demonstrates the heterogeneity of the studies in terms of characteristics [12]. Due to the heterogeneity of the studies in terms of the study type and outcomes across the studies, meta-analysis was not appropriate [12]. Costs were converted into British pounds sterling, £, using the appropriate exchange rate published in the International Monetary Fund [21] and inflated to 2017 price year using the hospital and community health services (HCHS) index [22–25] for the studies included in the review.

## Results

### Literature search: identification of studies

**Fig. 1:**
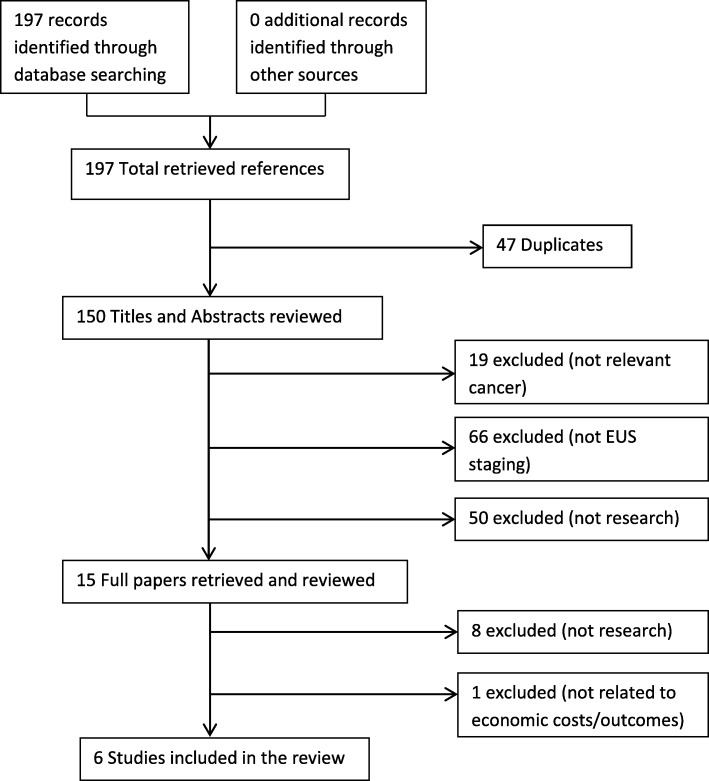
Flowchart of the study selection process

Overall, the search from 1996 to 2016 identified 197 potentially relevant studies, six of which fulfilled the inclusion criteria and were included in the review (Fig. 1). Of the six studies included, three were economic analysis studies and three were economic modelling studies.

To ensure that the review was as up-to-date as possible, the searches were re-run on all databases to cover 2016–2018 (search update on 28/04/2018); 30 potentially relevant papers were identified but none met the inclusion criteria. In such case, the final number of studies included in the review remained at six.

### Study descriptions

Tables 3 and 4 summarises the characteristics of the six studies included in the review. There were three economic analysis studies (Table 3) and three economic modelling studies (Table 4). Five of the studies included in the review were US studies, and one was a UK study. Of the three economic analysis studies, two were cost analyses [26, 27] and one was a cost-effectiveness analysis [11]. Of the three economic modelling studies, two were cost-minimisation analyses [29, 30] and one was a cost-effectiveness analysis [31]. All of the three economic modelling studies used decision tree modelling techniques to explore staging strategies.

**Table 3:** Summary table of the structure of the three economics papers included in the review

Authors, year, country	Aims of the study	Type of participants (*n*)	Type of study, methodology	Study perspective	Price year, currency (unit)	Type of intervention / staging technique	Method of delivery	Length of follow-up	Cost of intervention / staging technique	Type of economic analysis conducted	Outcomes / results / conclusions^a^
Shumaker et al. (2002) [26], USA.	To determine (1) the relative proportions of each oesophageal cancer stage in a group of patients referred for preoperative staging with EUS, (2) the proportion of patients with EUS stage 1 and 4 tumours that would not be treated with combined modality therapy, and (3) to estimate the potential cost savings of performing preoperative EUS in oesophageal cancer patients.	Patients with oesophageal cancer receiving preoperative staging with EUS (*n* = 180, 82% men and mean age 66.5 years).	Cost analysis using a retrospective review of a large multicentre national computerised endoscopic database. Data between February 1998 and October 2000 were extracted, reviewed and analysed.	Not stated specifically, the authors described US Medicare data	Price year: 2000 Currency: US dollars (USD$)	NA: retrospective review of a large national endoscopic database.	NA	NA	The cost of EUS for preoperative staging of oesophageal cancer was estimated at $634 per patient (£697 per patient, 2017 price year)	Cost analysis study: the potential cost savings of performing preoperative EUS in oesophageal cancer patients.	Preoperative staging of oesophageal cancer with EUS can facilitate cost savings by reducing the need for additional treatments in stage 1 and 4 oesophageal cancer (a significant proportion of patients – 26% in this series).
Chang et al. (2003) [27], USA	To determine the impact of EUS combined with FNA on patients’ choice of therapy and on the cost of care.	Patients diagnosed with oesophageal cancer (squamous-cell or adenocarcinoma) who were referred to the University of California’s Irvine Medical Center for preoperative EUS staging between August 1993 and August 1997 (*n* = 60, 39 men, 21 women and mean age 68 ± 10 years). These patients were all being considered for surgical resection and had undergone standard evaluation including CT which showed no evidence of distant metastases.	Cost analysis alongside prospective case series.	Not stated specifically, the study was undertaken in California, USA.	Not stated specifically, the authors described their cost analyses were based on the published direct costs of endosonography-guided aspiration biopsy and thoracotomy published in 1997 (Gress et al., 1997). Currency: US dollars (USD$)	NA: cost analysis study alongside prospective case series.	NA	Based on the data used in the cost analyses, the length of follow-up was, on average, 17 months (range 1–51 months).	The cost of EUS-FNA biopsy based on the published direct costs of endosonography-guided aspiration biopsy (Gress et al., 1997) was estimated at $1975 per patient (outpatient) (£3528 per patient, 2017 price year).	Cost analysis study: the cost of care for these patients was calculated to explore whether or not the use of EUS decreases the cost of managing patients with oesophageal cancer.	Patients’ decisions on whether to undergo medical or surgical treatment correlated significantly with their overall tumour staging, suggesting that the information provided by EUS played a significant role in patients’ decision-making. EUS-guided therapy potentially reduces the cost of managing patients with oesophageal cancer by USD$12,340 per patient (£10,510 per patient, 2017 price year) due to reduced number of thoracotomies undertaken (patient choice).
Russell et al. (2013) [11], UK	To examine whether the addition of EUS to usual staging uses resources cost-effectively.	Patients with proven cancer of the oesophagus, stomach or gastro-oesophageal junction; medically fit for both surgery (even if not planned) and chemotherapy, free of metastatic disease and had not started treatment. Both their ASA (America Society of Anesthesiologists) grading and their WHO performance status had to be 1 or 2 (*n* = 213, 165 male; mean age 64.4 years; EUS group (*n* = 107); No EUS group (*n* = 106)).	Cost-effectiveness analysis alongside a multi-centre randomised controlled trial (RCT) namely ‘COGNATE trial’. The study explored whether giving EUS scan in addition to standard staging algorithms would be more cost-effective compared to standard staging algorithms.	NHS perspective, focusing on health-care resources used by participants including investigation, treatment and palliation, and other elements of secondary and pharmaceutical care.	Price year 2008 Currency: Pounds Sterling (£)	Cancer staging with EUS vs. without EUS	Patients randomised to intervention group received EUS scan in addition to standard staging algorithms. Patients randomised to control group received standard staging algorithms.	Study follow-up period was 54 months or until death, whichever came first. Main analyses of the study (including health economic analysis) used 48 months.	The cost of EUS scan was £551 (day case) (£648, 2017 price year), £1477 (outpatient) (£1737, 2017 price year) and £3781 (inpatient) (£4447, 2017 price year).	Cost-effectiveness analysis using QALY as a measure of effect – The difference in cost and QALY between intervention and control groups was calculated; the probabilities of the EUS intervention being cost-effective at different willingness-to-pay thresholds were estimated.	EUS reduced net use of health-care resources by £2860 (£3364, 2017 price year) and had an increase of 0.1969 in estimated mean QALYs. Combining these estimated benefits and savings yields probability of 96.6% that EUS is cost-effective in the sense of achieving the NICE criterion of costing less than £20,000 to gain a QALY [28].

*NA* Not applicable, *ICER* incremental cost-effectiveness ratio, *EUS* endoscopic ultrasound, *EUS-FNA* endoscopic ultrasound-fine needle aspiration, *NHS* national health service, *QALY* quality-adjusted life year, *NICE* National Institute for Health and Care Excellence

^a^Converted to pound sterling (£) at 2017 prices

**Table 4:** Summary table of the structure of the three economic modelling papers included in the review

Authors, year, country	Aims of the study	Type of participants	Type of study, methodology	Perspective of the model	Price year, currency (unit)	Type of intervention / staging technique	Analysis	Time horizon	Outcome measure(s)	Outcomes / results / conclusions^a^
Hadzijahic et al. (2000) [29], USA	To determine whether it is less costly to request CT or EUS first to identify advanced oesophageal cancer; to determine which variables most affect the overall cost of identifying advanced disease.	Oesophageal cancer patients who underwent both CT and EUS between July 1995 and April 1999 (*n* = 124, mean age = 62.7 years, 98 (79%) men, and 72 (58%) white).	Cost-minimisation study using decision tree model to compare which of the two initial staging strategies (EUS first or CT first strategy) would cost less to detect advanced disease in patients diagnosed endoscopically with oesophageal cancer.	Not stated specifically, the study took local referral centre perspective.	Price year: 1999 Currency: US dollars (USD$).	CT first strategy vs. EUS first strategy.	Decision analysis using decision tree model.	Not stated specifically.	Overall cost of identifying advanced disease of the two strategies: EUS first and CT first strategies.	Initial CT is the least costly strategy if the probability of finding advanced disease by initial CT is greater than 20%, if the probability of finding advanced disease by initial EUS is less than 30%, or if the cost of EUS is greater than 3.5 times the cost of CT. EUS found advanced disease more frequently than CT (44% vs. 13%; *p* < 0.0001) and initial EUS was the least costly strategy (Initial EUS strategy expected cost was US$804 (£824, 2017 price year) vs. initial CT strategy expected cost $844 (£867, 2017 price year)).
Harewood et al. (2002) [30], USA	To examine which staging/management technique was the least costly: EUS FNA, CT-guided FNA or surgical management of oesophageal tumours.	Patients with apparently “resectable” oesophageal cancer on CT (i.e. patients with non-metastatic oesophageal cancer).	Cost-minimisation study using decision tree model to determine which strategy is least costly among the different alternatives: CT-FNA, EUS-FNA and ‘proceed straight to surgery’ options.	Third party payer perspective.	Price year: 2001 Currency: US dollars (USD$).	CT-FNA vs. EUS-FNA vs. ‘proceed directly to surgery’.	Decision analysis using decision tree model.	Not stated specifically.	Least costly staging strategy among the three strategies (CT-FNA vs. EUS-FNA vs Surgery)	EUS FNA was the least costly strategy at $13,811 (£14,578, 2017 price year), followed by surgery at $13,992 (£14,768, 2017 price year) and CT-FNA at $14,350 (£15,147, 2017 price year). EUS FNA remained the least costly option, provided that the prevalence of celiac lymph node (CLN) involvement was greater than 16%. Below this value, surgery became the least costly strategy. The final outcome of the model was also sensitive to variation in the sensitivity of EUS FNA. Provided that the sensitivity of EUS-FNA was greater than 66%, EUS-FNA remained the least costly staging option in the management of oesophageal tumours. Despite changing the values of two or three variables simultaneously in the two- and three-way sensitivity analyses, the result still showed that EUS FNA remained the least costly strategy.
Wallace et al. (2002) [31], USA	To compare the health care costs and effectiveness of multiple staging options for patients with oesophageal cancer.	All Medicare-eligible patients whose invasive oesophageal cancer was diagnosed between January 1991 and December 1996. Data were obtained retrospectively from the SEER–Medicare databases.	Cost-effectiveness study using decision tree model to compare the costs and effectiveness of six strategies (CT alone vs. CT + EUS vs. CT + TL vs. CT + EUS + TL vs CT + PET+EUS vs. PET+EUS).	Third-party payer perspective	Price year: 2000 Currency: US dollars (USD$).	The costs and effectiveness of the six strategies were compared – CT alone vs. CT + EUS vs. CT + TL vs. CT + EUS + TL vs CT + PET+EUS vs. PET+EUS.	Decision analysis using decision tree model.	Not stated specifically	Cost, QALYs and cost per QALY of the six strategies	Under baseline assumptions, CT + EUS-FNA was the least costly strategy and offered more QALYs, on average, than all other strategies with the exception of PET+EUS-FNA. The latter was slightly more effective but also more costly. The marginal cost-effectiveness ratio comparing PET+EUS-FNA with CT + EUS-FNA was $60,544 per QALY (£66,588 per QALY, 2017 price year). These findings were robust and changed very little in all of the sensitivity analyses.

*ICER* incremental cost-effectiveness ratio, *EUS* endoscopic ultrasound, *EUS-FNA* endoscopic ultrasound-fine needle aspiration, *CT* computed tomography, *PET* positron emission tomography, *TL* thoracoscopy and laparoscopy, *QALY* quality-adjusted life year

^a^Converted to pound sterling (£) at 2017 prices

The six studies included in the review differed quite markedly in terms of their design. Only one study used primary cost and outcome data collected in prospective evaluation [11], one study used data collected in prospective case series [27], one study used retrospective data [26], and the remaining three studies synthesised data from secondary sources in a decision tree model [29–31]. Of the six studies, only one [11] was a randomised controlled trial and included participants diagnosed with gastro-oesophageal cancer (i.e. oesophageal, gastro-oesophageal junction or gastric cancer); the other five were non-trial studies and included participants diagnosed with oesophageal cancer. Amongst the six studies, Russell et al. (2013) [11] was again the only study which evaluated costs of health care resource use covering secondary care contacts and hospital prescribed drugs in addition to cost of EUS, collected prospectively in the trial.

In terms of health outcome measures, two studies [11, 31] included quality-adjusted life year (QALY) as the measure of effect and conducted a cost-effectiveness analysis to assess the gain in QALYs relative to the costs of different staging strategies. The remaining four studies [26, 27, 29, 30] did not explore QALY or other quality of life measures but only cost.

### Quality assessment

Each of the six studies included in the review were critically appraised against the appropriate source of quality appraisal checklist: the CASP economic evaluation checklist [19] was used for the three economic studies, and Philips et al’s economic modelling checklist [20] was used for the remaining three economic modelling studies. Table 5 and Table 6 summarised the quality assessment of the three economic studies and three economic modelling studies, respectively.

**Table 5:** Quality assessment results of economic studies included in the systematic review

Question no.	CASP economic evaluation checklist questions^ab^	Response (√, x, NC or NA)		
		Studies (author and year)		
		Shumaker et al. (2002) [26]	Chang et al. (2003) [27]	Russell et al. (2013) [11]
1	Was a well-defined question posed?	√	√	√
2	Was a comprehensive description of the competing alternatives given?	NA	NA	√
3	Does the paper provide evidence that the programme would be effective (i.e. would the programme do more good than harm)?	√	√	√
4	Were the effects of the intervention identified, measured and valued appropriately?	NA	NA	√
5a	Were all important and relevant resources required and health outcome costs for each alternative identified?	NC	NC	√
5b	Were all important and relevant resources required and health outcome costs for each alternative measured in appropriate units?	√	√	√
5c	Were all important and relevant resources required and health outcome costs for each alternative valued credibly?	√	NC	√
6	Were costs and consequences adjusted for different times at which they occurred (discounting)?	x	x	√
7	What were the results of the evaluation?	√	√	√
8	Was an incremental analysis of the consequences and cost of alternatives performed?	NA	NA	√
9	Was an adequate sensitivity analysis performed?	√	x	√
10	Is the programme likely to be equally effective in your context or setting?	√	√	√
11	Are the costs translatable to your setting?	x	x	√
12	Is it worth doing in your setting?	√	√	√
Score, ratio™ (%)		8/11 (73%)	6/11 (55%)	14/14 (100%)

*NA* Not Applicable, *NC* Not Clear

^a^[19] Available from: http://www.casp-uk.net/casp-tools-checklists

^b^Adapted from: Drummond MF, Stoddart GL, Torrance GW. Methods for the economic evaluation of health care programmes. Oxford: Oxford University Press, 1987

™Ratio = b/a, where b = sum of tick; a = sum of items (excluding ‘NA’ items)

**Table 6:** Quality assessment results of economic modelling studies included in the systematic review

Quality Criterion	Philips et al’ economic modelling checklist questions^a^	Response (√, x, NC or NA)		
		Studies (author and year)		
		Hadzijahic et al. (2000) [29]	Harewood et al. (2002) [30]	Wallace et al. (2002) [31]
S1	Is there a clear statement of the decision problem?	√	√	√
	Is the objective of the evaluation and model specified and consistent with the stated decision problem?	√	√	√
	Is the primary decision-maker specified?	NC	√	√
S2	Is the perspective of the model stated clearly?	x	√	√
	Are the model inputs consistent with the stated perspective?	NC	√	√
	Has the scope of the model been stated and justified?	√	√	√
	Are the outcomes of the model consistent with the perspective, scope and overall objective of the model?	√	√	√
S3	Is the structure of the model consistent with a coherent theory of the health condition under evaluation?	√	√	√
	Are the sources of data used to develop the structure of the model specified?	√	√	√
	Are the causal relationships described by the model structure justified appropriately?	NA	NA	NA
S4	Are the structural assumptions transparent and justified?	√	√	√
	Are the structural assumptions reasonable given the overall objective, perspective and scope of the model?	√	√	√
S5	Is there a clear definition of the options under evaluation?	√	√	√
	Have all feasible and practical options been evaluated?	√	√	√
	Is there justification for the exclusion of feasible options?	NA	NA	NA
S6	Is the chosen model type appropriate given the decision problem and specified causal relationships within the model?	√	√	√
S7	Is the time horizon of the model sufficient to reflect all important differences between options?	x	x	X
	Are the time horizon of the model, the duration of treatment and the duration of treatment effect described and justified?	x	x	X
S8	Do the disease states (state transition model) or the pathways (decision tree model) reflect the underlying biological process of the disease in question and the impact of interventions?	√	√	√
S9	Is the cycle length defined and justified in terms of the natural history of disease?	NA	NA	NA
D1	Are the data identification methods transparent and appropriate given the objectives of the model?	√	NC	√
	Where choices have been made between data sources, are these justified appropriately?	NA	√	√
	Has particular attention been paid to identifying data for the important parameters in the model?	√	√	X
	Has the quality of the data been assessed appropriately?	x	x	x
	Where expert opinion has been used, are the methods described and justified?	NA	NA	x
D2	Is the data modelling methodology based on justifiable statistical and epidemiological techniques?	√	NC	√
D2a	Is the choice of baseline data described and justified?	√	√	√
	Are transition probabilities calculated appropriately?	NA	NA	NA
	Has a half-cycle correction been applied to both cost and outcome?	NA	NA	NA
	If not, has this omission been justified?	NA	NA	NA
D2b	If relative treatment effects have been derived from trial data, have they been synthesised using appropriate techniques?	NA	NA	NA
	Have the methods and assumptions used to extrapolate short-term results to final outcomes been documented and justified?	NA	NA	NA
	Have alternative assumptions been explored through sensitivity analysis?	√	√	√
	Have assumptions regarding the continuing effect of treatment once treatment is complete been documented and justified?	NA	NA	NA
	Have alternative assumptions regarding the continuing effect of treatment been explored through sensitivity analysis?	NA	NA	NA
D2c	Are the costs incorporated into the model justified?	√	√	√
	Has the source for all costs been described?	√	√	√
	Have discount rates been described and justified given the target decision-maker?	NC	NA	√
D2d	Are the utilities incorporated into the model appropriate?	NA	NA	√
	Is the source for the utility weights referenced?	NA	NA	X
	Are the methods of derivation for the utility weights justified?	NA	NA	X
D3	Have all data incorporated into the model been described and referenced in sufficient detail?	NC	√	√
	Has the use of mutually inconsistent data been justified (i.e. are assumptions and choices appropriate)?	NC	NC	√
	Is the process of data incorporation transparent?	√	x	X
	If data have been incorporated as distributions, has the choice of distribution for each parameter been described and justified?	NA	NA	NA
	If data have been incorporated as distributions, is it clear that second order uncertainty is reflected?	NA	NA	NA
D4	Have the four principal types of uncertainty been addressed?	x	x	X
	If not, has the omission of particular forms of uncertainty been justified?	x	x	X
D4a	Have methodological uncertainties been addressed by running alternative versions of the model with different methodological assumptions?	x	x	X
D4b	Is there evidence that structural uncertainties have been addressed via sensitivity analysis?	x	x	X
D4c	Has heterogeneity been dealt with by running the model separately for different subgroups?	x	x	x
D4d	Are the methods of assessment of parameter uncertainty appropriate?	√	√	√
	If data are incorporated as point estimates, are the ranges used for sensitivity analysis stated clearly and justified?	NC	√	√
C1	Is there evidence that the mathematical logic of the model has been tested thoroughly before use?	x	x	x
C2	Are any counterintuitive results from the model explained and justified?	NA	NA	NA
	If the model has been calibrated against independent data, have any differences been explained and justified?	NA	NA	NA
	Have the results of the model been compared with those of previous models and any differences in results explained?	x	x	x
Score, ratio™ (%)		21/38 (55%)	24/38 (63%)	28/43 (65%)

*NA* Not Applicable, *NC* Not Clear

^a^Available from [20]: Philips Z, Ginnelly L, Sculpher M, Claxton K, Golder S, Riemsma R, Woolacott N and Glanville J. Review of guidelines for good practice in decision-analytic modelling in health technology assessment. *Health Technol Assess* 2004;8(36)

™Ratio = b/a, where b = sum of tick; a = sum of items (excluding ‘NA’ items)

Table 5 shows the study quality of the three economic studies was generally good, scoring on average greater than 75%, although only one study [11] met all quality criteria on the CASP economic evaluation checklist. The study by Shumaker et al. (2002) [26] scored the second highest, followed by Chang et al. (2003) [27]. Of these three economic studies, two had missing key information: Chang et al. (2003) [27] reported neither cost perspective, cost inflation, discounting nor price year, and sensitivity analysis was not undertaken; likewise, Shumaker et al. (2002) [26] did not state whether their reported costs were discounted or inflated as appropriate.

Table 6 shows the study quality of the three economic modelling studies included in the review was satisfactory, scoring moderately well on the Philips et al’s economic modelling checklist. In descending order of quality, the study by Wallace et al. (2002) [31] scored the highest followed by Harewood et al. (2002) [30] and Hadzijahic et al. (2000) [29]. One study [29] did not state the perspective of the model and all three [29–31] did not specify the time horizon of the decision tree model. There was insufficient detail of how parameters in the model were identified [31] and how data were modelled [30]. There was also a lack of clarity with regards to the source of probabilities and cost data used in the decision tree model [29].

### Data synthesis results

All of the six studies included in the review exhibit EUS as a complementary imaging technique to other imaging modalities such as CT and PET scanning for staging gastro-oesophageal cancer. This is in agreement with a previously published meta-analysis study of diagnostic test characteristics for EUS, CT, and PET scanning [8], concluding that the three approaches were complementary.

Results from three of the economic studies [11, 26, 27] show staging of oesophageal or gastro-oesophageal cancer with EUS could potentially save costs. Similarly, results from two of the modelling studies [29, 30] show that EUS or EUS-fine-needle aspiration biopsy (FNA) is the least costly staging technique for oesophageal cancer. The study by Wallace et al. (2002) [31] shows that EUS-FNA in addition to CT scan is the least costly strategy than all other strategies i.e. CT alone, CT+ thoracoscopy and laparoscopy (TL), CT + EUS-FNA + TL, CT + PET+EUS-FNA and PET+EUS-FNA.

Results from the two studies [11, 31] in which quality-adjusted life year (QALY) and cost data were available demonstrate the use of EUS [11] or EUS-FNA [31] as an additional staging technique for gastro-oesophageal cancer offered more QALYs and costed less, on average, compared to staging techniques without EUS. Russell et al. (2013) [11] reported that EUS resulted in a QALY gain of 0.1969 QALYs and saved costs by £2860, on average, per patient (£3364 per patient, 2017 price year); combining these benefits and savings demonstrates that EUS is likely to be cost-effective with a probability of 96% at the UK NICE’s threshold of £20,000–£30,000 per QALY [28].

Similarly, Wallace et al. (2002)‘s [31] modelling study showed that using EUS-FNA as an additional staging technique offered greater QALYs and saved more costs, on average, than staging strategy without EUS. For example, the combination of CT and EUS-FNA (CT + EUS-FNA) provided 0.0019 more QALYs and saved US$1790, on average, per patient (£1969 per patient, 2017 price year) compared to CT alone strategy. The authors argued that, among all the six staging strategies evaluated (i.e. CT alone, CT + EUS-FNA, CT + TL, CT + EUS-FNA + TL, CT + PET+EUS-FNA and PET+EUS-FNA), CT + EUS-FNA was the least costly strategy (US$40,363) (£44,392, 2017 price year) and offered higher QALYs on average (0.9649) than all other strategies with the exception of PET+EUS-FNA (US$44,521 for 1.0336 QALYs) (£48,965, 2017 price year). The latter was slightly more effective (by 0.0687 QALYs on average) but more costly (by US$4158 on average [£4573, 2017 price year]) compared with CT + EUS-FNA, yielding a marginal cost-effectiveness ratio of US$60,544 per QALY (£66,588 per QALY, 2017 price year), a ratio that is less than that of other medical treatments but above accepted thresholds in the USA and UK.

## Discussion

### Main findings

This systematic review of economic evidence of EUS staging in patients with GOC revealed a considerably small number of relevant studies. Studies varied in quality, study design and method. Study quality was generally satisfactory across all the studies included in the review, but only one of these studies [11] met all reporting and quality criteria. Differences in study design make it difficult to draw definitive conclusions as to whether the use of EUS as an additional staging technique could be considered cost-effective i.e. value for money which can be assessed by comparing the costs (monetary term) and health effects (non-monetary term) of an intervention with the alternative. Health effect of an intervention is usually measured in terms of QALYs (Quality-Adjusted Life Years), a summary measure of health outcome and also a common unit used for economic evaluation of an intervention, as recommended by the UK’s NICE (The National Institute for Health and Care Excellence) [28]. Given the differences in study design, a head-to-head comparison of the results couldn’t be made from the Russell et al. (2013) [11] and Wallace et al. (2002) [31] studies to draw definitive conclusions. Although both of these studies had evaluated both costs and QALYs, their respective study designs were too different to allow direct comparison; one was an economic evaluation study using primary data [11] and the other an economic modelling study using secondary data [31]. Nevertheless, the economic evidence identified in this review, especially the better quality studies, provided useful findings on the value of EUS staging in the management of GOC patients, which could be of importance to policymakers and healthcare commissioners.

Among the six studies included in the review, two studies [11, 31] are the most robust in terms of including and comparing the relative costs and QALYs of different staging strategies, for example GOC staging with and without EUS. Findings from both of these two studies demonstrated that use of EUS as an additional imaging technique could save costs and offer greater QALY gains. This could be due to the fact that EUS has been known to be beneficial in terms of its sensitivity for locoregional staging of GOC [4, 6, 8, 32–34]. For that reason, using EUS as a complementary imaging technique to other imaging techniques such as CT and PET scanning for staging GOC could undoubtedly help minimise unnecessary treatments [4, 35, 36]; and thus potentially could save costs and offer greater health benefits to patients in terms of QALY gains. The EUS cost saving evidence was also supported by the remaining four studies [26, 27, 29, 30] evaluating only the cost of EUS e.g. whether EUS is a cost saving strategy or the least costly staging strategy. Russell et al. (2013) [11] further argued that EUS has a considerably high probability of being cost-effective under current recommended UK NICE’s threshold of £20,000 to £30,000 per QALY [28]. Thus, despite the scarcity of economic evidence in this field, from these studies identified in the review, there is some positive economic evidence relating to the cost-effectiveness of EUS in the management of patients with GOC.

### Strength and limitations of review methods

This review adds to the literature by providing critical evaluation of the health economics evidence of EUS staging in gastro-oesophageal cancers (GOCs), for which there is a lack of well-conducted economic studies. Though a systematic review in this field was published 20 years ago [37], this systematic review is the most up-to-date collection of economic literature in this area. Twenty years on since the review by Harris et al. (1998) [37], still only six papers were found in the area of health economics of EUS staging in GOC. This shows that there is a lack of prioritisation of research in this area.

Broad search terms were used to develop a comprehensive search strategy for each of the databases used in this systematic review. The resultant retrieved studies were quality appraised, using both the published standard checklists recommended for use in assessing the quality of economics articles in systematic review – the Critical Appraisal Skills Programme (CASP) economic evaluation checklist [19] and the Philips et al’s economic modelling checklist [20] for the retrieved economic studies and economic modelling studies, respectively. The narrative summary of the review not only described the economic evidence of EUS staging in GOC but also served as a platform for providing a holistic insight into the health economics research available to date in this area. The latter is particularly helpful for commissioners, clinicians and researchers to elicit information and potentially to facilitate the development of further research in this area.

This review has several limitations. Heterogeneity of the included studies in the review in terms of study designs and methods meant that a meta-analysis of studies was not possible and a narrative summary was used. We also acknowledge that different countries have different thresholds for both the investigation of, and surgical management of gastro-oesophageal malignancies. This can result in variation between practices and hence difficulty in translating financial recommendations across regions. In terms of impact that EUS has on patients’ quality of life and its costs, the lack of the availability of health economics research in this area means that it is considerably difficult, particularly for commissioners and clinicians, to guide evidence-based practice from an economic perspective.

### Further research

This systematic review shows that the economic evidence available to date in this area is still scarce. There was a lack of health economic research collecting data, especially primary data, on both costs and effects (such as utility values to construct QALYs) of EUS staging in GOC. To improve this, there is a need for more primary health economic research in this area, particularly integrated clinical and economic trials of EUS staging in GOC that can offer robust evidence of costs and effects.

## Conclusions

Despite the lack of economic evidence on costs and benefits of EUS staging for GOC, the data available from this review suggest use of EUS as a complementary staging technique to other staging techniques for GOC appears to be cost saving and offers greater QALYs. Based on the only randomised controlled trial conducted in the UK identified in this review, EUS seems to have high probability of being cost-effective at the UK NICE’s threshold of £20,000–£30,000 per QALY. Nevertheless, future studies are necessary because the economic evidence around EUS staging interventions for GOC is far from robust. More health economic research and good quality data are needed to judge the economic benefits of EUS staging for GOC, particularly primary health economic research that collects primary data on the costs and effects (such as QALYs) of EUS staging in GOC.

## Additional file

Additional file 1: An example of search strategy used in the Medline Ovid database. Medline ovid search strategy for the systematic review (DOCX 18 kb)

## Data Availability

Available data was presented in the main manuscript. And, one additional file was generated.

## Abbreviations

CASP: Critical Appraisal Skills Programme
CRUK: Cancer Research United Kingdom
CT: Computer tomography
EUS: Endoscopic ultrasound
GOCs: Gastro-oesophageal cancers
MRI: Magnetic resonance imaging
PET: Positron emission tomography
RCT: Randomised controlled trials
