# Supplementary material for: Endoscopic ultrasound staging in patients with gastro-oesophageal cancers: a systematic review of economic evidence
Source: BMC Cancer. 2019 Sep 9;19:900. doi: 10.1186/s12885-019-6116-0 (PMC6734454; doi:10.1186/s12885-019-6116-0)
Supplement: Supplementary file 1 — An example of search strategy used in the Medline Ovid database. Medline ovid search strategy for the systematic review (DOCX 18 kb) [file 12885_2019_6116_MOESM1_ESM.docx]

**Additional file 1.** An example of search strategy used in the Medline Ovid database

| 1 | exp Endosonography/ |
| --- | --- |
| 2 | endosono$.tw. |
| 3 | endoscopic ultraso$.tw. |
| 4 | endoscopic-ultraso$.tw. |
| 5 | EUS.tw. |
| 6 | (echoendoscop$ or echo-endoscop$).tw. |
| 7 | ((endosono$ or endoscopic ultraso$ or endoscopic-ultraso$ or EUS) adj6 aspiration).tw. |
| 8 | 1 or 2 or 3 or 4 or 5 or 6 or 7 |
| 9 | staging.tw. |
| 10 | ((Preoperative or pre-operative) adj6 staging).tw. |
| 11 | 9 or 10 |
| 12 | 8 and 11 |
| 13 | exp Adenocarcinoma/ |
| 14 | adenocarcinoma$.tw. |
| 15 | 13 or 14 |
| 16 | exp Esophagus/ |
| 17 | exp Esophagogastric Junction/ |
| 18 | (gastroesophag$ adj3 junction$).tw. |
| 19 | (gastro-esophag$ adj3 junction$).tw. |
| 20 | (gastrooesophag$ adj3 junction$).tw. |
| 21 | (gastro-oesophag$ adj3 junction$).tw. |
| 22 | esophagogastric junction$.tw. |
| 23 | esophago-gastric junction$.tw. |
| 24 | oesophagogastric junction$.tw. |
| 25 | oesophago-gastric junction$.tw. |
| 26 | exp Stomach/ |
| 27 | 16 or 17 or 18 or 19 or 20 or 21 or 22 or 23 or 24 or 25 or 26 |
| 28 | 15 and 27 |
| 29 | exp Esophageal Neoplasms/ |
| 30 | exp Stomach Neoplasms/ |
| 31 | (esophag$ adj5 neoplas$).tw. |
| 32 | (oesophag$ adj5 neoplas$).tw. |
| 33 | (esophag$ adj5 cancer$).tw. |
| 34 | (oesophag$ adj5 cancer$).tw. |
| 35 | (esophag$ adj5 carcin$).tw. |
| 36 | (oesophag$ adj5 carcin$).tw. |
| 37 | (esophag$ adj5 tumo$).tw. |
| 38 | (oesophag$ adj5 tumo$).tw. |
| 39 | (esophag$ adj5 metasta$).tw. |
| 40 | (oesophag$ adj5 metasta$).tw. |
| 41 | (esophag$ adj5 malig$).tw. |
| 42 | (oesophag$ adj5 malig$).tw. |
| 43 | (esophag$ adj5 adenocarcinoma$).tw. |
| 44 | (oesophag$ adj5 adenocarcinoma$).tw. |
| 45 | (stomach adj5 neoplas$).tw. |
| 46 | (stomach adj5 cancer$).tw. |
| 47 | (stomach adj5 carcin$).tw. |
| 48 | (stomach adj5 tumo$).tw. |
| 49 | (stomach adj5 metasta$).tw. |
| 50 | (stomach adj5 malig$).tw. |
| 51 | (stomach adj5 adenocarcinoma$).tw. |
| 52 | (gastric adj5 neoplas$).tw. |
| 53 | (gastric adj5 cancer$).tw. |
| 54 | (gastric adj5 carcin$).tw. |
| 55 | (gastric adj5 tumo$).tw. |
| 56 | (gastric adj5 metasta$).tw. |
| 57 | (gastric adj5 malig$).tw. |
| 58 | (gastric adj5 adenocarcinoma$).tw. |
| 59 | 29 or 30 or 31 or 32 or 33 or 34 or 35 or 36 or 37 or 38 or 39 or 40 or 41 or 42 or 43 or 44 or 45 or 46 or 47 or 48 or 49 or 50 or 51 or 52 or 53 or 54 or 55 or 56 or 57 or 58 |
| 60 | 28 or 59 |
| 61 | (gut$ adj5 (neoplas$ or cancer$ or carcin$ or tumo$ or adenocarcinoma$ or metasta$ or malig$)).tw. |
| 62 | (gullet$ adj5 (neoplas$ or cancer$ or carcin$ or tumo$ or adenocarcinoma$ or metasta$ or malig$)).tw. |
| 63 | (food pipe adj5 (neoplas$ or cancer$ or carcin$ or tumo$ or adenocarcinoma$ or metasta$ or malig$)).tw. |
| 64 | (("upper GI" or "upper-GI") adj5 (neoplas$ or cancer$ or carcin$ or tumo$ or adenocarcinoma$ or metasta$ or malig$)).tw. |
| 65 | (("upper gastrointestin$" or "upper-gastrointestin$") adj5 (neoplas$ or cancer$ or carcin$ or tumo$ or adenocarcinoma$ or metasta$ or malig$)).tw. |
| 66 | ((upper digestive tract$ or upper-digestive tract$) adj5 (neoplas$ or cancer$ or carcin$ or tumo$ or adenocarcinoma$ or metasta$ or malig$)).tw. |
| 67 | 61 or 62 or 63 or 64 or 65 or 66 |
| 68 | 60 or 67 |
| 69 | 12 and 68 |
| 70 | exp Economics/ |
| 71 | health economics.mp. |
| 72 | Economic evaluation.mp. |
| 73 | exp Cost-Benefit Analysis/ |
| 74 | (cost$ adj2 (effective$ or utilit$ or consequence$ or benefit$ or minimi$)).tw. |
| 75 | Cost effectiveness analysis.mp. |
| 76 | cost utility analysis.mp. |
| 77 | cost consequences analysis.mp. |
| 78 | cost minimisation analysis.mp. |
| 79 | cost minimization analysis.mp. |
| 80 | exp "Costs and Cost Analysis"/ |
| 81 | (unit cost or unit-cost or unit-costs or unit costs or drug cost or drug costs or hospital costs or health-care costs or health care cost or medical cost or medical costs).tw. |
| 82 | (cost$ adj2 (efficac$ or analys$ or allocation$ or control$ or illness$ or affordable$ or fee$ or charge$)).tw. |
| 83 | exp Models, Economic/ |
| 84 | (decision adj1 (tree$ or analys$ or model$)).tw. |
| 85 | Markov$.tw. |
| 86 | exp Economics, Pharmaceutical/ or exp Economics, Medical/ or exp Economics, Hospital/ |
| 87 | (econom$ or cost$ or price$ or pricing or pharmacoeconomic$ or pharmaeconomic$ or pharmaco-economic$).tw. |
| 88 | exp "Fees and Charges"/ |
| 89 | exp Budgets/ |
| 90 | (financ$ or fee$).tw. |
| 91 | ((value or values or valuation) adj2 (money or monetary or life or lives or costs or cost)).tw. |
| 92 | exp Health Expenditures/ |
| 93 | (low adj cost).mp. |
| 94 | (high adj cost).mp. |
| 95 | (health?care adj cost$).mp. |
| 96 | (cost adj estimate$).mp. |
| 97 | exp Hospital Costs/ |
| 98 | exp "Cost Savings"/ |
| 99 | exp "Quality of Life"/ |
| 100 | *"Quality of Life"/ |
| 101 | 70 or 71 or 72 or 73 or 74 or 75 or 76 or 77 or 78 or 79 or 80 or 81 or 82 or 83 or 84 or 85 or 86 or 87 or 88 or 89 or 90 or 91 or 92 or 93 or 94 or 95 or 96 or 97 or 98 or 99 or 100 |
| 102 | 69 and 101 |
